# Exploring the use of AI-generated counterfactual chest X-rays to enhance diagnostic learning in medical education

**DOI:** 10.1186/s12909-026-09597-7

**Published:** 2026-06-12

**Authors:** Greta Mohr, Yifei Zhu, Xujiong Ye, Marilyn Lennon, Calum MacLellan, John Maclay, David J. Lowe, Christopher Sainsbury, Feng Dong, David Lagnado

**Affiliations:** 1 University College London, London, UK; 2 University of Strathclyde, Glasgow, UK; 3 University of Exeter, Exeter, UK; 4 NHS Greater Glasgow and Clyde, Glasgow, UK

**Keywords:** Artificial Intelligence, Counterfactual Images, Radiology Education, Diagnostic Learning, Chest X-rays

## Abstract

Accurate interpretation of chest X-rays is a critical clinical skill, yet radiology training in medical education remains limited and often fails to provide broad exposure to a wide variety of conditions, including those that are rare, subtle, or easily confused with one another. Although artificial intelligence (AI) has shown impressive performance in medical image classification, its potential to actively improve clinical education has not been fully realised. In this study, we introduce AI-generated *counterfactual* chest X-rays—real patient images digitally altered to show a different but still realistic condition, while keeping the same patient’s anatomy. These counterfactuals create ‘what if’ scenarios within the same patient and offer a novel tool to enhance diagnostic learning, support decision-making, and improve confidence calibration in medical trainees and professionals.

Forty-two participants, including medical students and doctors, completed a four-part online study involving diagnostic classification, comparison of counterfactual image pairs, image authenticity judgements, and clinical treatment planning. All image comparisons focused on three clinical conditions: healthy lungs, pneumonia, and pleural effusion. Statistical analyses included t-tests, ANOVAs, Pearson correlations, and linear regression.

Results showed that counterfactual images meaningfully supported diagnostic learning. Participants demonstrated improved accuracy and confidence over time, particularly when distinguishing pleural effusion from healthy lungs. Confidence became more closely aligned with accuracy, and participants were better able to recognise condition-specific differences. These comparisons also revealed areas of diagnostic weakness that would be difficult to detect through conventional instruction alone. While real images led to higher raw diagnostic accuracy, counterfactuals proved highly effective in promoting learning and reflective reasoning. Image authenticity influenced clinical decision-making, and initial confidence was a stronger predictor of final treatment confidence than diagnostic correctness.

These findings highlight the strong potential of counterfactual imaging to enhance radiology education. By enabling learners to engage with rare, complex, or demographically underrepresented cases in a controlled and consistent way, AI-generated counterfactuals offer a powerful and scalable tool to improve diagnostic preparedness and clinical confidence in future healthcare professionals.

**Supplementary Information:**

The online version contains supplementary material available at 10.1186/s12909-026-09597-7.

## Background

Chest X-rays are the most commonly conducted medical imaging procedures, and their accurate and prompt interpretation is crucial for ensuring high-quality patient care, generally analysed by radiological experts trained specifically in interpreting medical imaging. However, the growing workload of radiologists often results in non-radiologist physicians being tasked with interpreting medical images, even though they lack extensive radiology training [1–4], and so initial interpretation is often performed by the clinician directly involved in clinical care without the benefit of specialist interpretation and reporting. Non-radiologist physicians are often less accurate in interpreting chest X-rays [5, 6], considerably increasing the likelihood of misdiagnosis [7]. These difficulties apply not only to rare or subtle abnormalities but also to common conditions such as pneumonia and pleural effusion, which can still be misclassified without structured training.

Unfortunately, diagnostic radiology is often insufficiently emphasised in undergraduate medical education, leaving graduates feeling unprepared for accurate radiological interpretation in clinical practice [8, 9]. Radiology education is often inconsistent, and is typically integrated into other modules rather than being taught as a standalone subject [8, 10, 11], receiving only 5% of teaching time [8, 12, 13]. It is unsurprising then that many healthcare professionals struggle with accurately interpreting X-rays [14–19]. Together, these findings lend support for a more structured and dedicated curriculum, containing engaging and varied resources, to adequately prepare students for radiological tasks in their future medical practice [8, 9, 13, 19, 20].

Despite artificial intelligence (AI) models excelling in specific tasks like detecting abnormalities in medical images [21–23], with some models even performing at a level comparable to or surpassing experts [1], the complexity of real-world diagnoses requires clinical context and critical reasoning that AI systems cannot fully replicate. While AI has shown considerable promise in augmenting the diagnostic process, the continued need for well-trained radiologists and clinicians is undeniable and human expertise remains crucial in refining diagnostic decisions, especially in complex and ambiguous cases [24]. The challenges with AI lie in its inability to recognise nuances across diverse cases, particularly with rare or overlapping conditions, which may compromise diagnostic accuracy in certain situations [25] or the navigation situations where ethical and complex clinical judgement are required [26, 27]. Therefore, even with advanced AI, the role of skilled clinicians remains indispensable in providing high-quality, contextually informed care [25, 28].

Despite the promising advances in AI, its use in medical and radiological education remains limited [29]. However, there is growing recognition of the need to integrate AI into radiology training [30–32]. Together, these findings point to a valuable opportunity to develop more effective methods for radiology education by incorporating AI into existing medical training frameworks [26, 33]. Here, we focus on leveraging AI not as a tool for making diagnostic predictions, but rather as a means to generate enhanced educational resources, namely counterfactual images, that can enrich the learning experience for radiologists by enabling the systematic exploration of rare, complex, or ambiguous conditions [34].

A counterfactual image in the medical context refers to an AI-modified version of a real patient’s medical image, where specific features are altered to represent a different pathological state, while maintaining the patient’s anatomical and demographic consistency. Grounded in the principles of causal inference, this approach simulates what a patient’s image *would have looked like* had a different disease or condition manifested, enabling clinicians to reason about alternative outcomes. For example, a counterfactual image might take a chest X-ray from a patient diagnosed with pneumonia and computationally modify it to show how the same lungs would have appeared had the patient instead been healthy, or had they developed a pleural effusion. This within-patient comparison allows learners to isolate and recognise the radiological features that distinguish one condition from another. This approach offers two key advantages: (1) it allows for the creation of more varied datasets by synthesising more images of underrepresented conditions and (2) it enables the examination of within-patient changes, allowing the visualisation of different conditions within the same individual. This causal counterfactual generation provides a richer and more flexible learning environment, preparing clinicians to recognise a wide array of diagnostic challenges both across populations and within individual patients.

In the current study, we explore the integration of AI-generated counterfactual chest X-rays into medical training. We aim to establish whether exposure to synthetic, between- and within-patient images can aid in skill development and improve diagnostic accuracy and confidence. Additionally, we evaluate clinicians’ ability to detect whether images are real or AI-generated. Finally, we investigate the influence of AI-generated images on treatment planning, examining whether participants’ clinical decisions are affected by the perceived authenticity of the images. Illustrative examples of the generated counterfactual images, alongside their corresponding real chest X-rays, are provided in Supplementary Figure S1. Overall, our study aims to provide a deeper understanding of how AI-generated chest X-rays can be effectively integrated into medical education and practice.

## Methods

### Participants and procedure

The study sample consisted of 42 participants, aged between 20 and 67 years (M = 26.1, SD = 7.4, 24 Female, 18 Male). Participants were recruited through Prolific (https://www.prolific.com). Participants were either medical students (*N* = 26) or medical doctors (*N* = 16). For students, years of medical experience included time in medical school, whereas for doctors this reflected years of postgraduate clinical practice. Across the full sample, the combined mean experience (including study time) was 6.2 years (SD = 5.09). All participants provided informed consent prior to participation, and the study was approved by the University College London Institutional Review Board (IRB): 0487.

Participants were directed to the online experiment platform Gorilla (https://app.gorilla.sc/) and provided with an information sheet outlining the study, including the nature of the task, consent procedures, and data usage. Formal informed consent was obtained before participation. Participants were compensated £6 for their time, which took approximately one hour to complete.

**Fig. 1:**
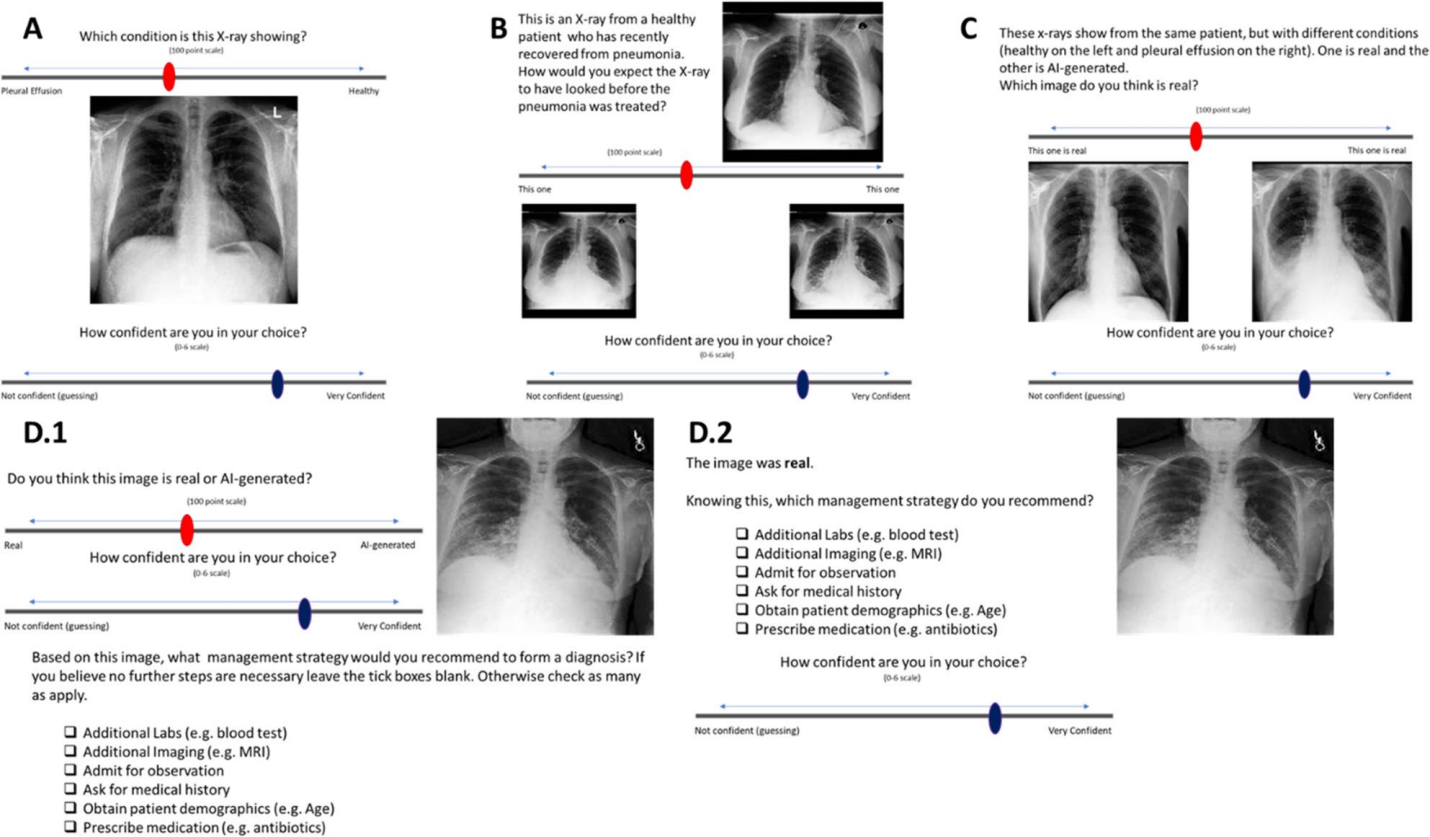
Schematic showing example trials across tasks. The schematic illustrates representative trials for each of the four tasks examined in this study: **A** Diagnostic Task—participants classify chest X-rays into one of three conditions (Healthy, Pneumonia, Pleural Effusion) based on the presented image; **B** Counterfactual Task—participants identify condition changes in paired X-rays while demographic and anatomical features remain consistent; **C** AI Identification Task—participants determine whether paired images (one real, one modified) are AI-generated or real; **D.1** & **D.2** Treatment Planning Task—participants select initial and revised treatment plans after identifying whether the X-ray image is AI-generated or real

The task was divided into four blocks. Each block consisted of a different task, and at the beginning of each block, participants were instructed what the task in the block would be and taken through a practice trial. Figure 1, below, shows a detailed schematic of the experimental design.

At the end of the study, participants were debriefed, informed of the full nature of the study, and assured that their data would be anonymised and stored securely. They were given the opportunity to ask any remaining questions and provided with contact details in case they wished to withdraw their data or inquire further.

#### Block 1- diagnostic task

The purpose of this block was to establish a baseline performance for basic diagnostic ability based on chest x-rays. This block consisted of 36 trials, separated into 2 parts (“pre” and “post”). For the first eighteen “pre” trials, participants were presented with a chest x-ray image showing one of 3 conditions; pneumonia, pleural effusion or healthy. Participants were then asked to rate on a100-point scale, which condition they believe is being shown, from an option of 2 choices (on each side of the scale)). The condition being shown, and the 2 options as well the location of the correct option, was varied and randomised across trials. Following the “pre” trials, participants were informed that in the following trials, the images they will be shown are a mix of genuine and AI-generated images. All other aspects of the task were the same for the eighteen “post” trials. Participants were then asked to rate how confident they were in their choice.

#### Block 2 - counterfactual learning

The purpose of this block was to explore how AI-generated images can be used as counterfactuals for prognostics. This block consisted of 18 trials. “For each trial, participants were shown a real chest X-ray from a patient with one of three conditions (Healthy, Pneumonia, or Pleural Effusion). They were then asked to select between two AI-generated counterfactual images of the same patient, each depicting one of the two other conditions. Their task was to identify which image corresponded to the designated target condition (i.e., the specific alternative diagnosis prompted in the trial). For example, in a Pneumonia → Healthy trial, the original image depicted Pneumonia, and participants were instructed to identify which of the two counterfactuals showed the patient as Healthy.” Participants were then asked to rate on 100-point scale, which of the two counterfactual images they believe is showing the matching x-ray, in this case ‘what the x-ray would have looked like while the patient was suffering from pneumonia’. Participants were then asked to rate how confident they are in their choice.

#### Block 3 - counterfactual AI identification task

The purpose of this block was to explore whether medical students and doctors are able to distinguish AI-generated images from real x-rays. This block consisted of 18 trials. On each trial, participants were shown two x-ray images from the same patient; one ‘real’ and one altered to show the same patient with a different condition (e.g. a ‘real’ pleural effusion x-ray image and an AI-generated pneumonia x-ray image from the patient).

#### Block 4 - treatment management task

The purpose of this block was to establish whether the source (AI-generated or real) of an image influences a treatment plans proposed by participants. In this block, participants were shown a series of x-ray images (again; healthy, pleural effusion or pneumonia) but not told which condition they are seeing. For each one, they were first asked to judge if they believed the image was AI-generated or real, followed by a confidence rating. They were then asked to create a management plan by selecting from a check box list which follow-ups they would recommend for this patient based on the x-ray image, which were generated by expert clinicians. The options were: *Additional Labs (e.g. blood test)*,* Additional Imaging (e.g. MRI)*,* Admit for observation*,* Ask for medical history*,* Obtain patient demographics (e.g. Age)*, and *Prescribe medication (e.g. antibiotics).* It was then revealed if the image was AI-generated or real and participants were asked again to select follow up treatments from the same check box list and provide a confidence rating.

### Task measures

#### Task accuracy

In this experiment, task accuracy refers to the participants’ ability to correctly distinguish between images of patients in different conditions; namely, Healthy, Pleural Effusion, or Pneumonia—and their ability to accurately identify whether an image is real or AI-generated. Participants used a 100-point slider, where each end represented one of the two possible answers. For each trial, participants rated the images on a scale from 0 (left image/answer) to 100 (right image/answer). In cases where the correct image/answer was positioned on the left, the scale was reversed, so that 0 corresponded to the correct answer. Effectively, this measure represents the mean slider distance from the correct answer.

This design allows for more granular feedback compared to a simple binary right–wrong approach, aligning with principles of educational assessment that emphasise capturing gradations of performance rather than dichotomous outcomes [35, 36]. By rewarding partial knowledge and penalising larger deviations, the slider measure functions similarly to confidence-weighted scoring methods, which have been shown to enhance diagnostic calibration and promote metacognitive monitoring in medical education [37, 38]. Such approaches encourage learners to reflect not only on the correctness of their decision but also on the strength of their evidence, which is a core component of self-regulated learning [39]. Unlike a straightforward right-or-wrong task, this method provides continuous feedback, motivating participants to refine their judgements and increase accuracy. The supplementary Table S1 provides a detailed comparison of decision strength and confidence across different conditions (Correct, Incorrect, and Uncertain) in the first and second halves of four blocks. The table highlights how decision strength, measured by the distance from the correct answer, varies for each trial type, alongside the participants’ reported confidence.

#### Self-reported confidence

Participants when then asked to rate how confident they are in their choice on a 0–6 scale where 6 is ‘very confident’ and 0 is ‘guessing’.

#### Treatment plan

The treatment plan task measure in your experiment involves participants selecting appropriate follow-up actions based on the x-ray images they were shown. After judging whether an image is real or AI-generated, participants were asked to choose from a list of six treatment/action options. These options could include actions or procedures relevant to the patient’s condition depicted in the x-ray.

The primary measure for this task is the number of treatments selected, which is quantified by summing the actions chosen by each participant. Additionally, the task also assesses changes in treatment selections after participants were informed whether the image was real or AI-generated. This provides insight into whether the knowledge of the image’s source (AI or real) influences participants’ treatment decisions.

#### Trial type

The trial type variable in this study refers to the categorical variable that defines the type of trial or comparison participants are making when assessing chest x-ray images. Specifically, it represents the different conditions participants are asked to evaluate, which involve distinguishing between various medical conditions (such as healthy, pleural effusion, and pneumonia) using real or AI-generated images. Each trial type corresponds to a specific comparison between two conditions (e.g., Healthy vs. Pleural Effusion, or Pleural Effusion vs. Pneumonia), with participants required to assess which image/answer corresponds to the correct diagnosis.

### Statistical analysis

The following MATLAB syntax was used to conduct the various statistical analyses in the experiment:

- Paired t-tests:

[h, p, ci, stats] = ttest(data.var1, data.var2).

- Pearson Correlation Analyses:

[r, p] = corrcoef(data.var1, data.var2).

- ANOVA:

[p, tbl, stats] = anovan(data.dependent_var, {data.group_var }, ‘model’, ‘model_type)

A premium subscription to ChatGPT using GPT4.0 was used to assist the analysis. All information provided by ChatGPT was critically evaluated and independently verified by the author. Code excerpts were tested to ensure syntactic and functional accuracy.

### Generation of stimuli

Counterfactual chest X-ray images were generated from the MIMIC-CXR [40] and MIMIC IV [41] datasets using a causal generative modelling framework designed to produce hypothetical visual variations that answer “what if” questions by altering underlying attributes (e.g., age, sex, or diagnosis). We focused on modifying images labelled as pneumonia, pleural effusion, or healthy to depict the other two conditions, allowing controlled comparisons across clinically relevant categories. N.B. any original images where the patient was diagnosed with both pleural effusion and pneumonia were not included in the study.

At the core of the method is a deep Structural Causal Model (SCM), which encodes assumed cause-effect relationships among variables. Image generation is handled by a Hierarchical Variational Autoencoder (HVAE), a deep generative model that reconstructs images through multiple layers of latent variables. Counterfactual inference follows a three-step process: abduction (inferring latent causes from the original image), intervention (modifying a selected causal factor), and prediction (generating the resulting counterfactual image).

To ensure medical plausibility, an integrated expert model evaluates the clinical coherence of generated images and guides refinement of the causal mechanisms. An active learning component further improves performance by selecting uncertain or underrepresented cases for retraining, enhancing generalisability and fidelity.

This integration of causal reasoning, deep generative modelling, expert validation, and adaptive data selection produces counterfactual X-rays that are both clinically relevant and causally consistent, suitable for use as experimental stimuli.

## Results

In an online study, participants (*N* = 42) consisting of medical students and medical doctors completed a 4-block task, throughout which they made diagnostic judgements based on real and AI-generated chest x-rays showing 3 conditions; healthy, pleural effusion and pneumonia.

### Block 1 - diagnostic task

In this block, participants were shown a series of chest X-ray images from patients who were either healthy, had pneumonia, or had a pleural effusion. Halfway through the block, participants were informed that the remaining trials would include a mix of real and AI-generated images; all other aspects of the task were unchanged.

**Fig. 2:**
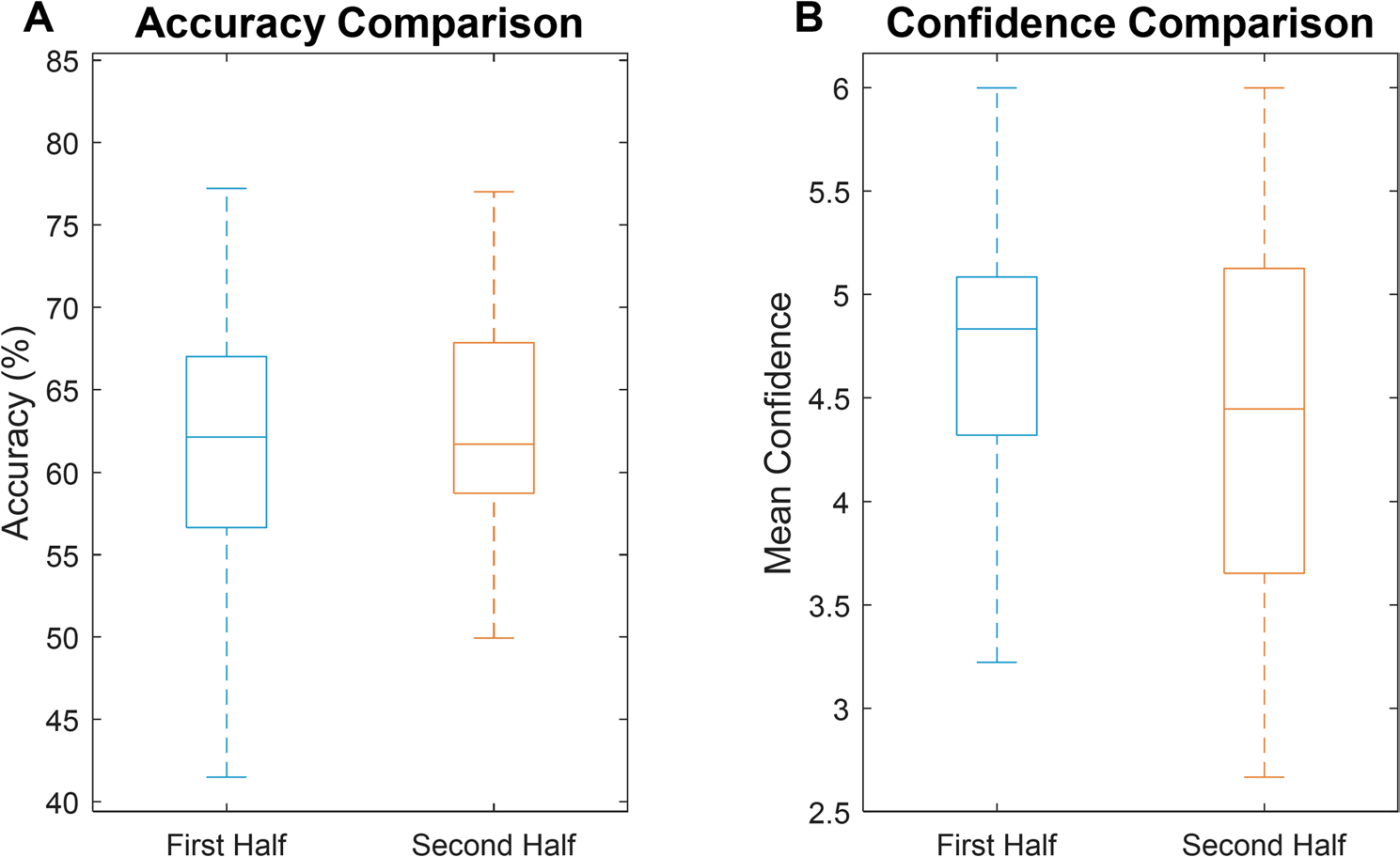
Box plots comparing Accuracy and Mean Confidence between the pre and post trials. **A** Distribution of accuracy across the pre (First Half) and post (Second Half) conditions. **B** Distribution of mean confidence across the same conditions. The box plots display the interquartile range (IQR), with the central line indicating the median value, and the whiskers extending to the range of the data. No outliers are shown

In the first half, participants performed moderately well, with a mean accuracy of 61.9% and relatively high confidence (M = 4.73). Accuracy in the second half was 1.1% higher than in the first half, although this difference was not significant (*t*(78) = 0.76, *p* = .45). Confidence decreased slightly by 0.25, but this difference was also not significant (*t*(79) = − 1.43, *p* = .15). Overall pre- vs. post-reveal accuracy and confidence distributions are shown in Fig. 2.

At the participant level, mean confidence was not significantly associated with mean accuracy in either half of the block. The overall correlation between confidence and accuracy was slightly negative and non-significant before the reveal (*r* = –.03, *p* = .85), shifting slightly positive after the reveal (*r* = .02, *p* = .91).

Correlations for each trial type are summarised in Table 1. As shown, none reached significance, although all shifted descriptively in the positive direction from pre- to post-reveal.

**Table 1:** Participant-level correlations between confidence and accuracy for the pre- and post-reveal phases of Block 1

Trial type	Pre-reveal *r* (*p*)	Post-reveal *r* (*p*)	Δr
EP	0.03 (0.85)	0.23 (0.15)	↑ 0.20
HE	0.13 (0.42)	0.13 (0.41)	– 0.00
PH	–0.24 (0.13)	0.00 (0.99)	↑ 0.24
Overall	–0.03 (0.85)	0.02 (0.91)	↑ 0.05

Note. Correlation coefficients are Pearson’s *r*. Δ*r* represents the change from pre- to post-reveal; arrows indicate direction. No correlations reached significance (*ps* > 0.05)

**Fig. 3:**
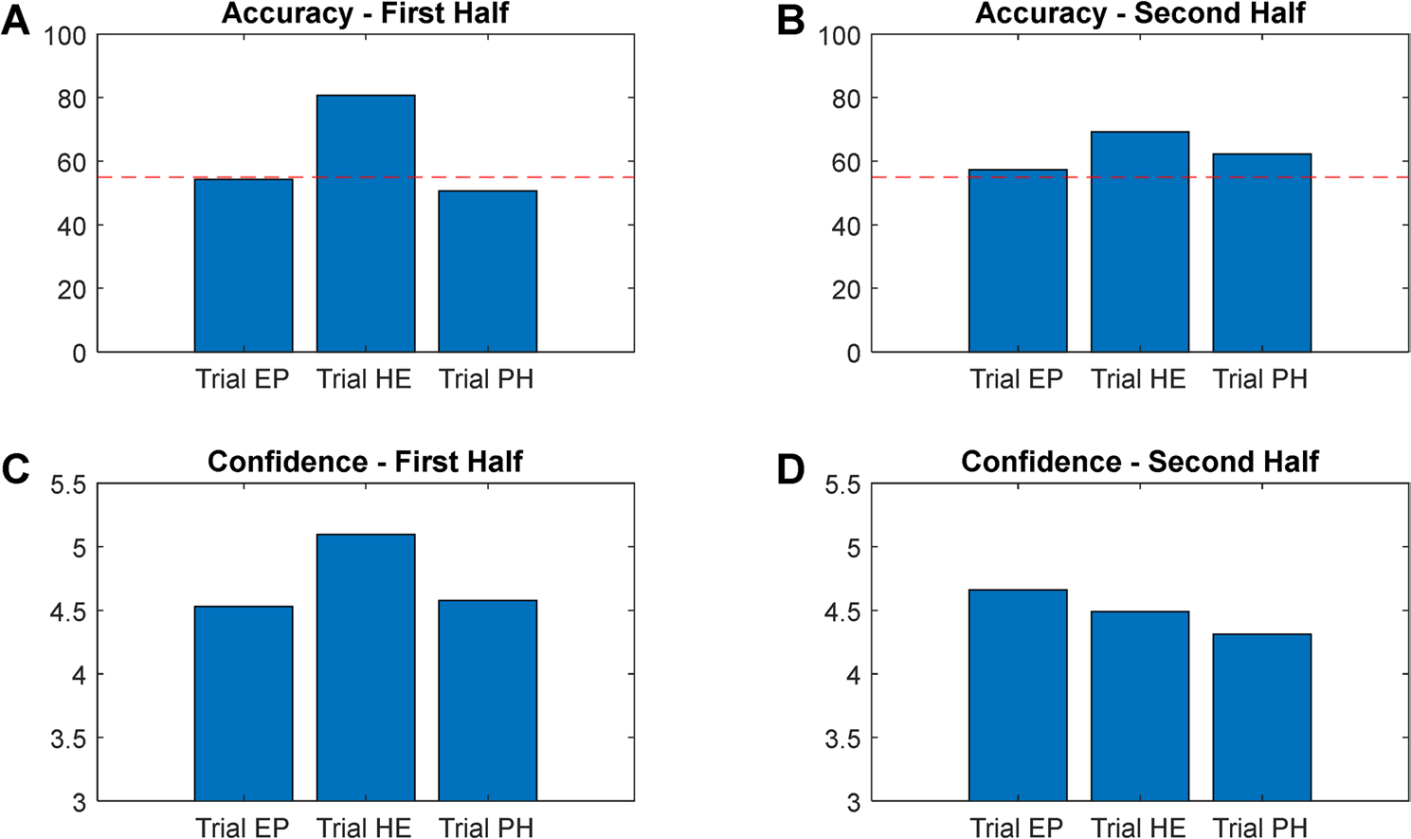
Bar charts comparing Accuracy and Confidence for the first and second half across trial types. **A** Mean accuracy across the pre (First Half) and (**B**) post (Second Half) of the block. **C** Mean confidence across the same trial types across the pre (First Half) and (**D**) post (Second Half) of the block

Despite no significant change in overall accuracy, participants performed above the predefined chance threshold (55%) in all three trial types in the second half (EP: 57.5%, HE: 69.2%, PH: 62.3%), which was not the case in the first half (EP: 54.3%, HE: 80.7%, PH: 50.6%) (shown in Fig. 3). Accuracy for HE trials decreased in the second half, which may reflect the inclusion of AI-generated images. A paired *t*-test showed higher accuracy for real versus AI-generated images, *t*(40) = − 6.20, *p* < .001. Confidence was also higher for real images than for AI-generated ones, *t*(40) = − 3.60, *p* < .001.

An ANOVA examining the effect of trial type on mean confidence revealed no significant differences, (*F*(2,122) = 1.36, *p* = .26), whereas accuracy differed significantly across trial types (*F*(2,122) = 9.18, *p* < .001). Post hoc comparisons showed that Healthy–Effusion trials were more accurate than both Effusion–Pneumonia (*t*(40) = 4.53, *p* < .001), and Pneumonia–Healthy, (*t*(40) = 2.53, *p* = .02). No significant difference was found between Effusion–Pneumonia and Pneumonia–Healthy, *t*(40) = − 1.68, *p* = .10.

### Block 2 - counterfactual learning

In this block, participants were shown a series of real chest X-ray images from patients who were either healthy, had pneumonia, or had a pleural effusion. They were then asked to make a counterfactual judgement between two AI-generated images of the same patient, representing the two remaining conditions. For example, in a P→H trial, the original image depicted pneumonia, and the task was to judge which of two AI-generated images showed the healthy condition.

Participants performed moderately well overall, with a mean accuracy of 67.0% and relatively high confidence (M = 4.68). At the participant level, there was no significant overall association between mean confidence and mean accuracy across the block (*r* = .03, *p* = .83). When broken down by trial type, correlations varied in strength: E→H (*r* = –.00, *p* = .98), E→P (*r* = –.04, *p* = .81), H→E (*r* = .12, *p* = .46), H→P (*r* = .39, *p* = .015), P→E (*r* = .29, *p* = .072), and P→H (*r* = .18, *p* = .26). Thus, only Healthy → Pneumonia trials showed a significant positive calibration effect, with Pneumonia → Effusion trials trending toward significance.

A one-way ANOVA revealed a significant effect of trial type on accuracy, *F*(5, 228) = 8.33, *p* < .001, indicating that participants’ counterfactual judgements varied depending on the conditions being compared. Post hoc comparisons showed that participants were significantly more accurate when distinguishing Healthy vs. Pneumonia compared to both Healthy vs. Effusion (*t*(466) = − 3.88, *p* < .001) and Pneumonia vs. Effusion (*t*(466) = − 3.65, *p* < .001). Accuracy did not differ significantly between Effusion vs. Healthy and Effusion vs. Pneumonia trials (*t*(466) = 0.16, *p* = .87).

**Fig. 4:**
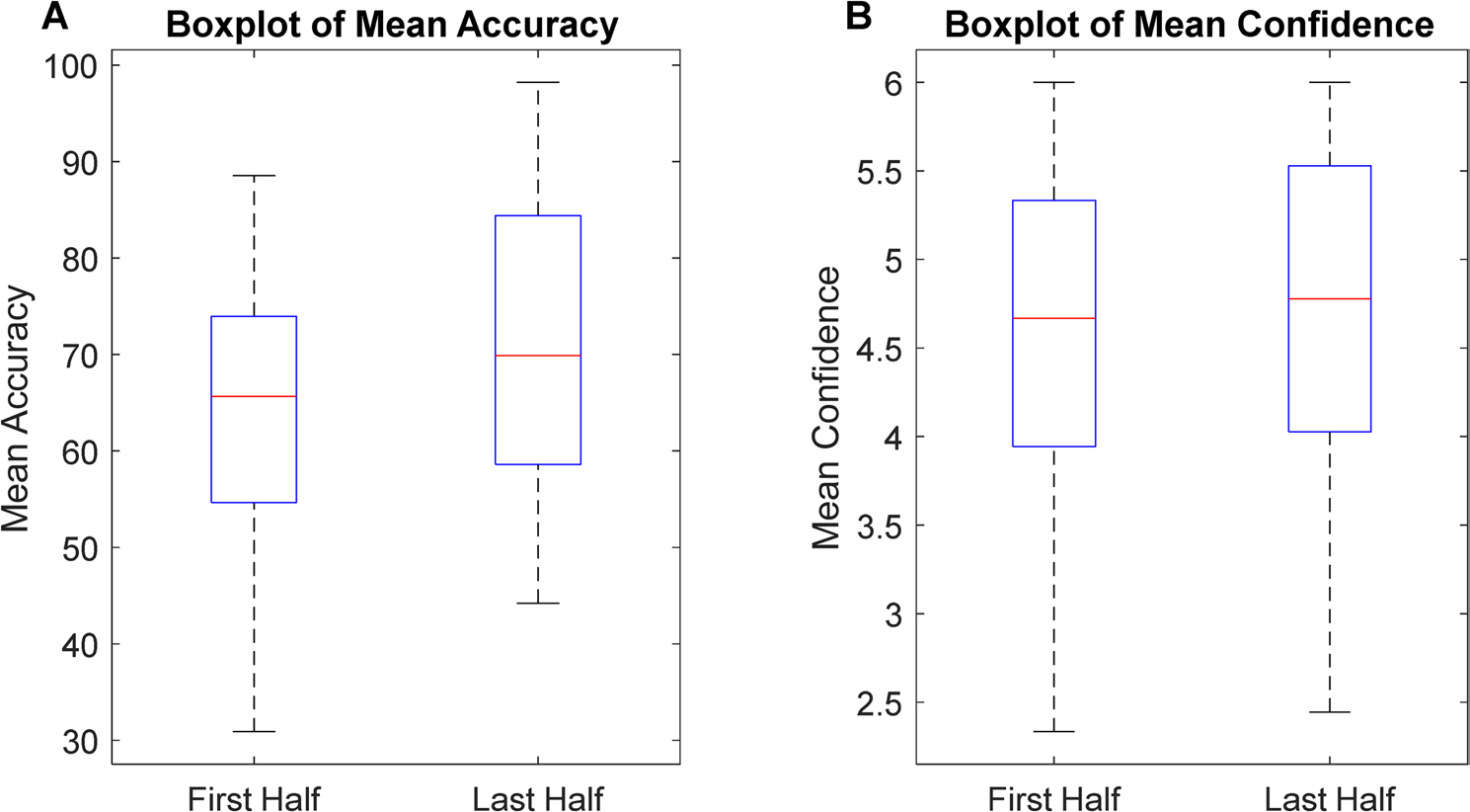
Box plots comparing Accuracy and Mean Confidence between the first and second half of the block. **A** Distribution of accuracy. **B** Distribution of mean confidence. The box plots display the interquartile range (IQR), with the central line indicating the median value, and the whiskers extending to the range of the data. No outliers are shown

To examine potential learning effects, the block was split into halves (Fig. 4). Accuracy improved by 6.8% from the first to the second half (*t*(76) = − 2.11, *p* = .04), whereas confidence did not change (*t*(76) = − 0.01, *p* = .99). Participant-level correlations showed weak calibration overall in both halves (first half: *r* = .01, *p* = .98; second half: *r* = .12, *p* = .45). Trial-type analyses are reported in Table 2.

**Table 2:** Participant-level correlations between confidence and accuracy by trial type for the first and second halves of Block 2

Trial type	First half *r* (*p*)	Second half *r* (*p*)	Δr
E→H	–0.04 (0.82)	0.12 (0.48)	↑ 0.16
E→P	0.17 (0.32)	0.14 (0.41)	↓ 0.03
H→E	0.11 (0.54)	0.12 (0.50)	↑ 0.01
H→P	**0.43 (0.012)**	0.17 (0.31)	↓ 0.26
P→E	0.22 (0.23)	0.16 (0.36)	↓ 0.06
P→H	0.01 (0.94)	0.13 (0.44)	↑ 0.12
Overall	0.01 (0.98)	0.12 (0.45)	↑ 0.11

Correlation coefficients are Pearson’s *r*. Δ*r* represents the change in correlation between the first and second halves; arrows indicate direction of change. Boldface indicates significant correlations at *p* < .05. Outliers are omitted

As shown in Table 2, the only significant calibration effect occurred in H → P trials in the first half (*r* = .43, *p* = .012), which did not persist in the second half. No other trial types demonstrated significant confidence–accuracy associations, though several shifted descriptively in the positive direction across halves.

Finally, accuracy changes between the first and second halves were examined separately for each trial type. Percentage increases were 3.11% for Healthy → Pneumonia, 10.65% for Effusion → Healthy, and 5.18% for Effusion → Pneumonia. However, none of these changes were statistically significant (H → P: *t*(232) = − 0.90, *p* = .37; E → H: *t*(232) = − 0.27, *p* = .78; E → P: *t*(232) = − 0.60, *p* = .55).

Figure 4. Box plots comparing accuracy and mean confidence between the first and second halves of Block 2. (A) Accuracy distribution. (B) Confidence distribution. Boxes represent the interquartile range (IQR), with medians indicated by horizontal lines; whiskers extend to the full range of the data.

### Block 3 - counterfactual AI identification task

In this block, participants were shown paired chest X-ray images from the same patient: one real and one altered to show a different condition. The conditions were healthy, pleural effusion, and pneumonia. Participants were informed which diagnosis each image depicted and asked to judge which of the two was real. For clarity, trial types are labelled according to the transition from the altered image to the real one (e.g., Effusion → Healthy [EH] means the altered image depicted pleural effusion and the real image was healthy). The purpose of this block was to explore whether medical students and doctors could distinguish AI-generated images from real X-rays. While this does not mirror clinical practice directly, it provides an important test of the realism and plausibility of counterfactuals for educational use.

Participants performed well, with a mean accuracy of 67.4% and relatively high confidence (M = 4.41). At the participant level, there was no overall association between confidence and accuracy across the block (*r* = –.01, *p* = .94). However, the pattern differed across trial types. Significant positive correlations were observed in Effusion → Healthy (EH) trials (*r* = .35, *p* = .025) and Pneumonia → Healthy (PH) trials (*r* = .37, *p* = .016), indicating that participants who were more accurate in these conditions also reported greater confidence. In contrast, Pneumonia → Effusion (PE) trials showed a significant *negative* correlation (*r* = –.50, *p* = .001), suggesting that participants were most confident precisely when they were least accurate. The remaining trial types, Effusion → Pneumonia (EP), Healthy → Effusion (HE), and Healthy → Pneumonia (HP), showed weak, non-significant relationships.

**Fig. 5:**
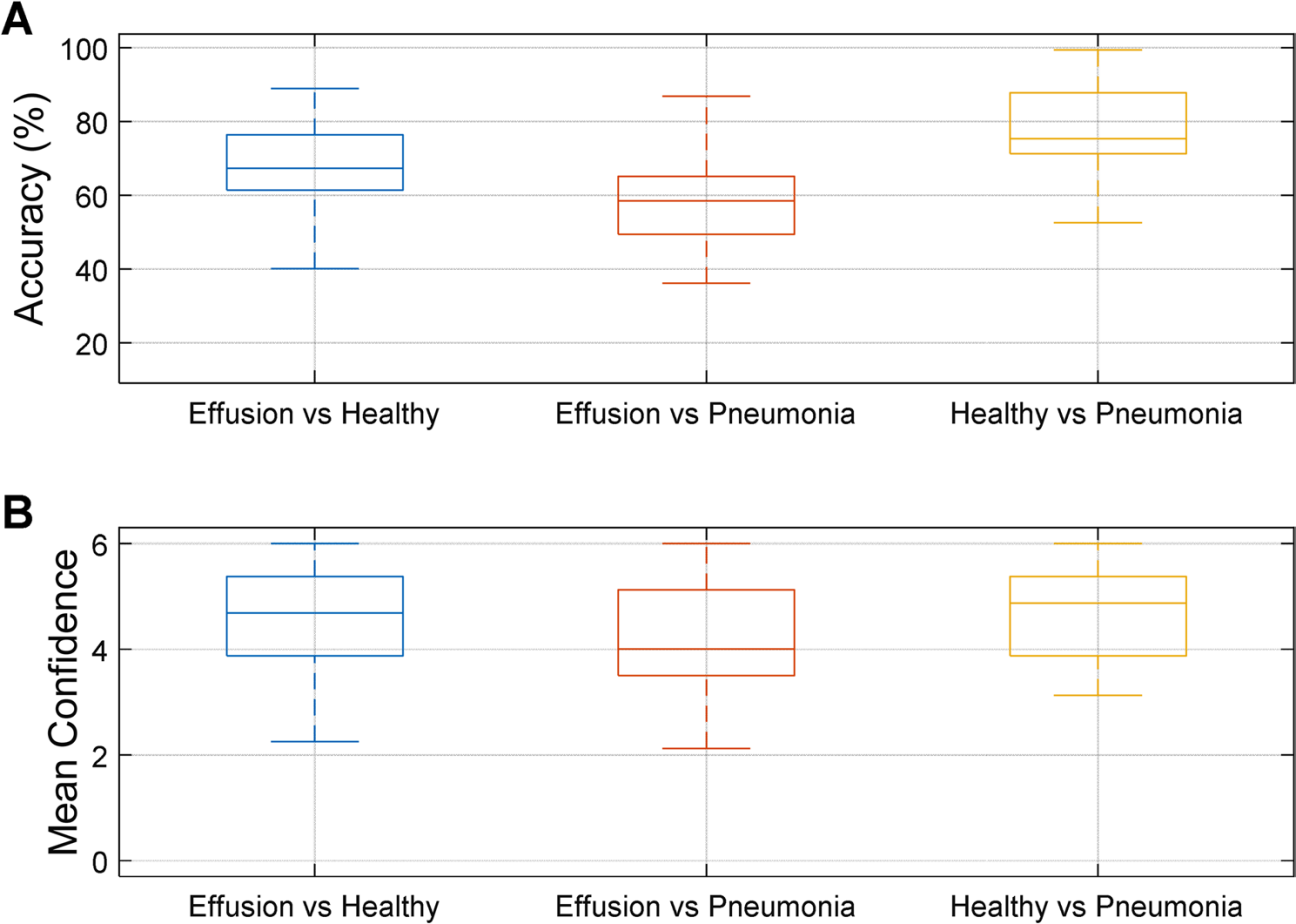
Box plots showing mean accuracy and mean confidence by trial type. **A** displays the mean accuracy distribution across different trial types. **B** shows the distribution of mean confidence ratings. The box in each plot represents the interquartile range (IQR), with the line inside the box indicating the median value

A one-way ANOVA revealed a significant effect of trial type on accuracy, *F*(2,123) = 21.8, *p* < .001, indicating that discrimination performance varied across conditions. Post hoc comparisons showed that participants were more accurate on Effusion–Healthy trials than on both Pneumonia–Effusion (*t*(82) = 3.62, *p* = .001) and Pneumonia–Healthy (*t*(82) = − 3.12, *p* = .003) trials. Accuracy was also higher on Pneumonia–Effusion than on Pneumonia–Healthy trials (*t*(82) = − 6.36, *p* < .001). In contrast, there was no significant effect of trial type on confidence, *F*(2,123) = 1.98, *p* = .11. Accuracy and confidence by trial type are shown in Fig. 5.

When comparing the first and second halves of the block, there was no significant change in confidence (*t*(125) = 0.17, *p* = .87) and no significant change in accuracy (*t*(125) = 1.06, *p* = .29), suggesting that performance remained stable over time.

Together, these findings indicate that while participants were generally able to identify real from altered X-rays above chance, their confidence–accuracy calibration varied considerably by trial type, with both strong positive and negative associations depending on the diagnostic contrast.

### Block 4 - treatment management task

In this block, participants were shown a series of chest x-ray images (real or AI-generated) depicting patients who were either healthy, with pleural effusion, or with pneumonia. They were told which diagnosis each image showed and asked to identify which image was real. After this identification task, participants provided an initial treatment plan, revised it following additional information, and finally rated their confidence in the final plan. The purpose of this block was to explore whether the source of an image (real or AI-generated) influenced participants’ management selections and confidence, rather than to evaluate the clinical correctness of treatment plans. Participants were asked to select from a set of possible management actions (e.g., ordering further tests, prescribing medication), and the primary measure was the number of actions chosen before and after the source was revealed. This was intended as an exploratory proof-of-concept task rather than a direct analogue to real-world clinical decision-making.

**Fig. 6:**
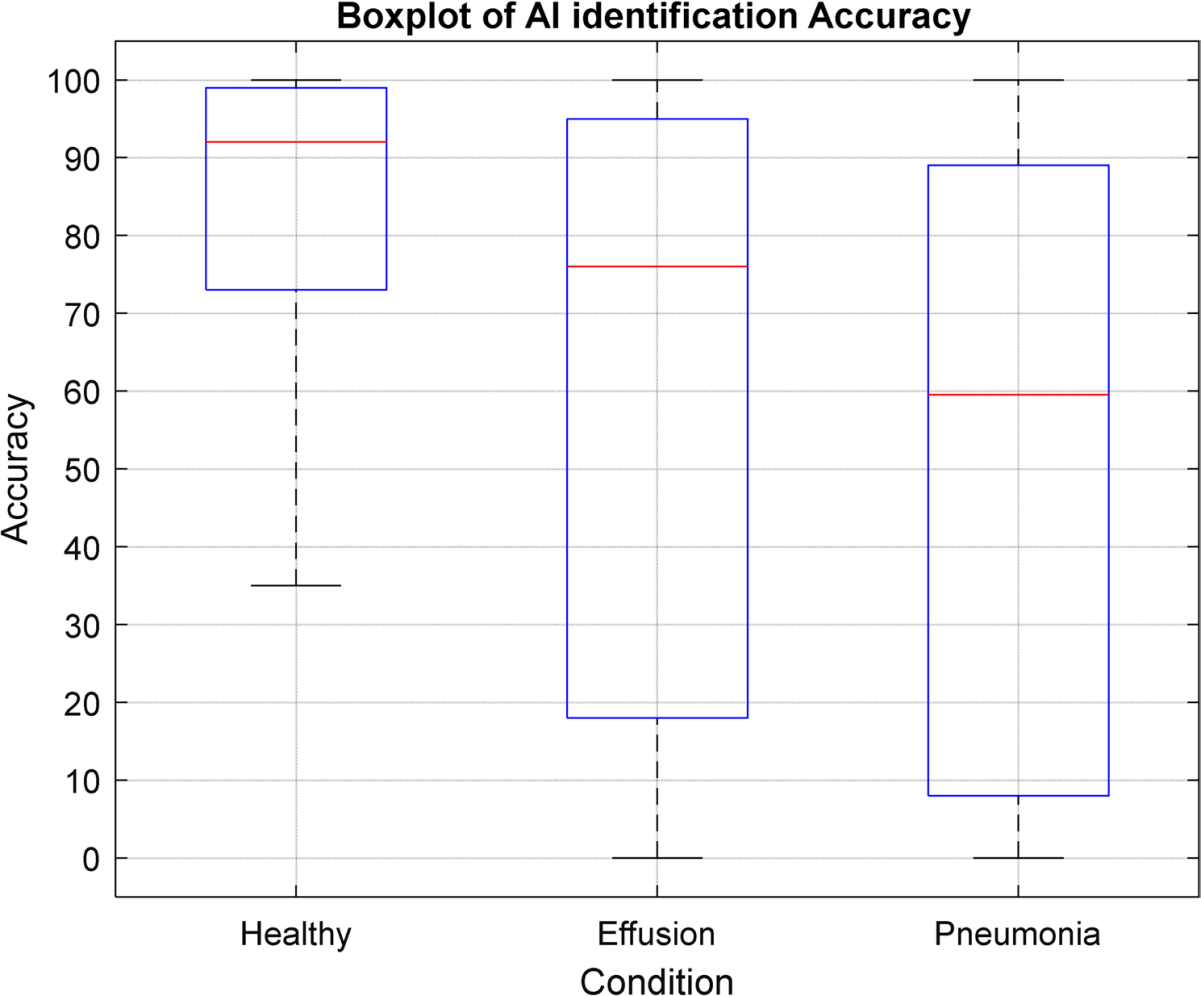
Boxplot showing the distribution of AI identification accuracy across different conditions. The x-axis represents the different conditions, and the y-axis shows the accuracy values. The boxplot illustrates the interquartile range (IQR) with the central line indicating the median accuracy, while the whiskers represent the spread of the data. Outliers are not shown in this plot

A 2 (Source: Real vs. AI) × 3 (Condition: Healthy, Effusion, Pneumonia) repeated-measures ANOVA on identification accuracy, with Participant as a random factor, revealed significant main effects of Condition, *F*(2,82) = 33.52, *p* < .001, and Source, *F*(1, 41) = 4.53, *p* = .039, qualified by a significant Condition × Source interaction, *F*(2,82) = 16.46, *p* < .001. Follow-up paired comparisons showed that the pattern of accuracy differences across conditions varied depending on whether the images were Real or AI-generated (Fig. 6).

For judgement confidence, there were significant main effects of Condition (*F*(2,82) = 8.80, *p* < .001), and Source (*F*(1, 41) = 12.28, *p* = .001), with no significant interaction. Participants were generally more confident in some conditions than others and more confident overall with Real images.

**Fig. 7:**
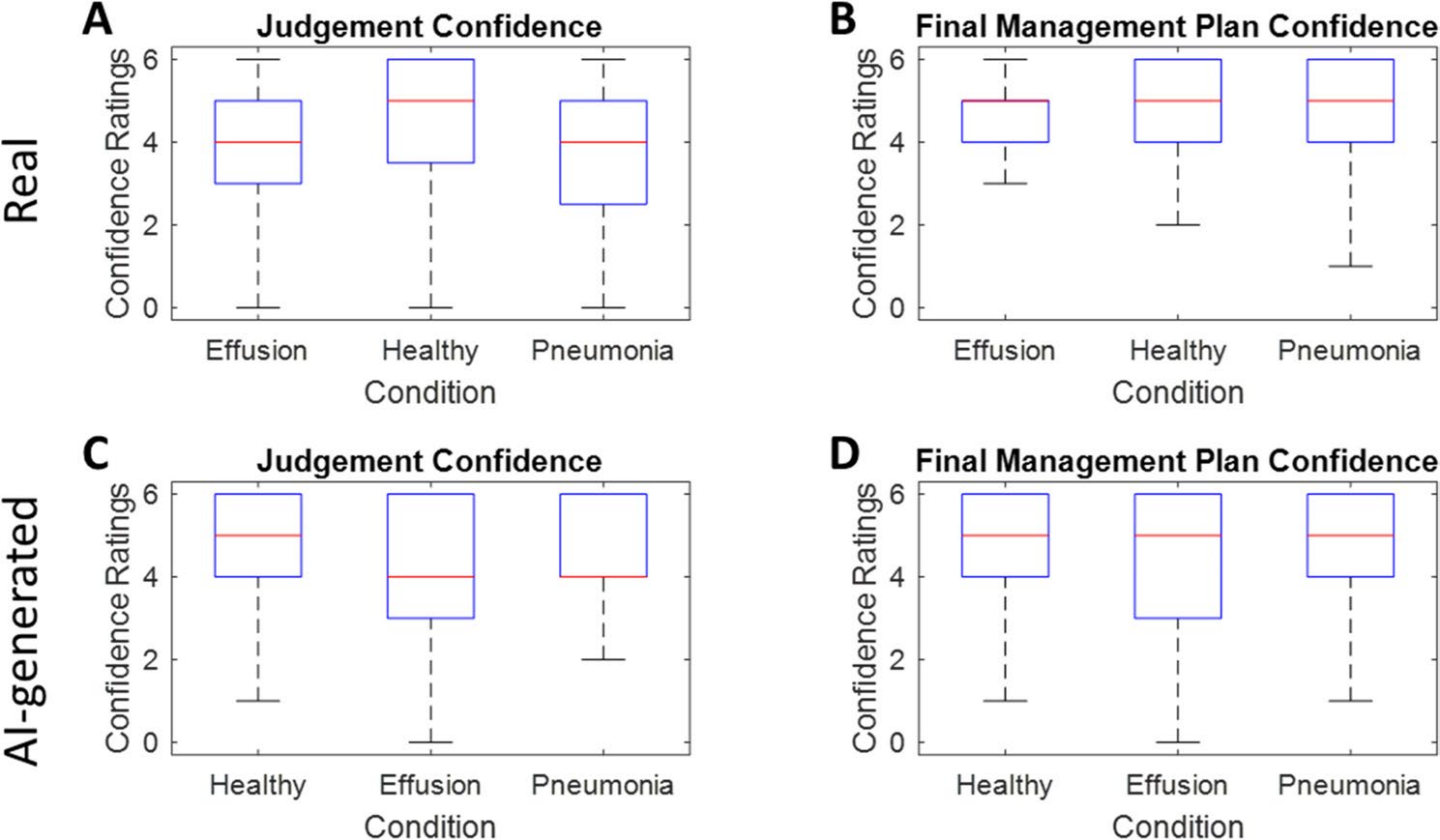
Box plots showing the distribution of confidence ratings for different conditions and source types. **A** Judgement Confidence and (**B**) Final Management Plan Confidence for real images. **C** Judgement Confidence and (**D**) Final Management Plan Confidence for AI-generated images

For final management plan confidence, no significant main effects or interactions were observed, all *p* > .18, indicating that final confidence ratings were relatively stable across conditions and sources. A paired t-test comparing confidence ratings before and after revision showed that participants were significantly more confident in their final plan than in their initial judgement (*t*(41) = 3.73, *p* = .001) (Fig. 7).

At the participant level, final management confidence was not reliably associated with identification accuracy overall (*r* = .19, *p* = .221), or when examined separately for Real (*r* = .12, *p* = .436) and AI (*r* = .19, *p* = .219) trials. This relationship was not significant when examined separately within Real or AI trials. This relationship was not significant when examined separately within Real (*r*(40) = − 0.00, *p* = .981) or AI (*r*(40) = − 0.19, *p* = .222) trials.

For the initial number of treatments chosen, the ANOVA revealed a significant main effect of Condition, (*F*(2,82) = 18.39, *p* < .001), and a Condition × Source interaction, (*F*(2,82) = 3.32, *p* = .04). This suggests that treatment selection strategies differed across conditions, and that the effect varied depending on whether the case was Real or AI-generated.

**Fig. 8:**
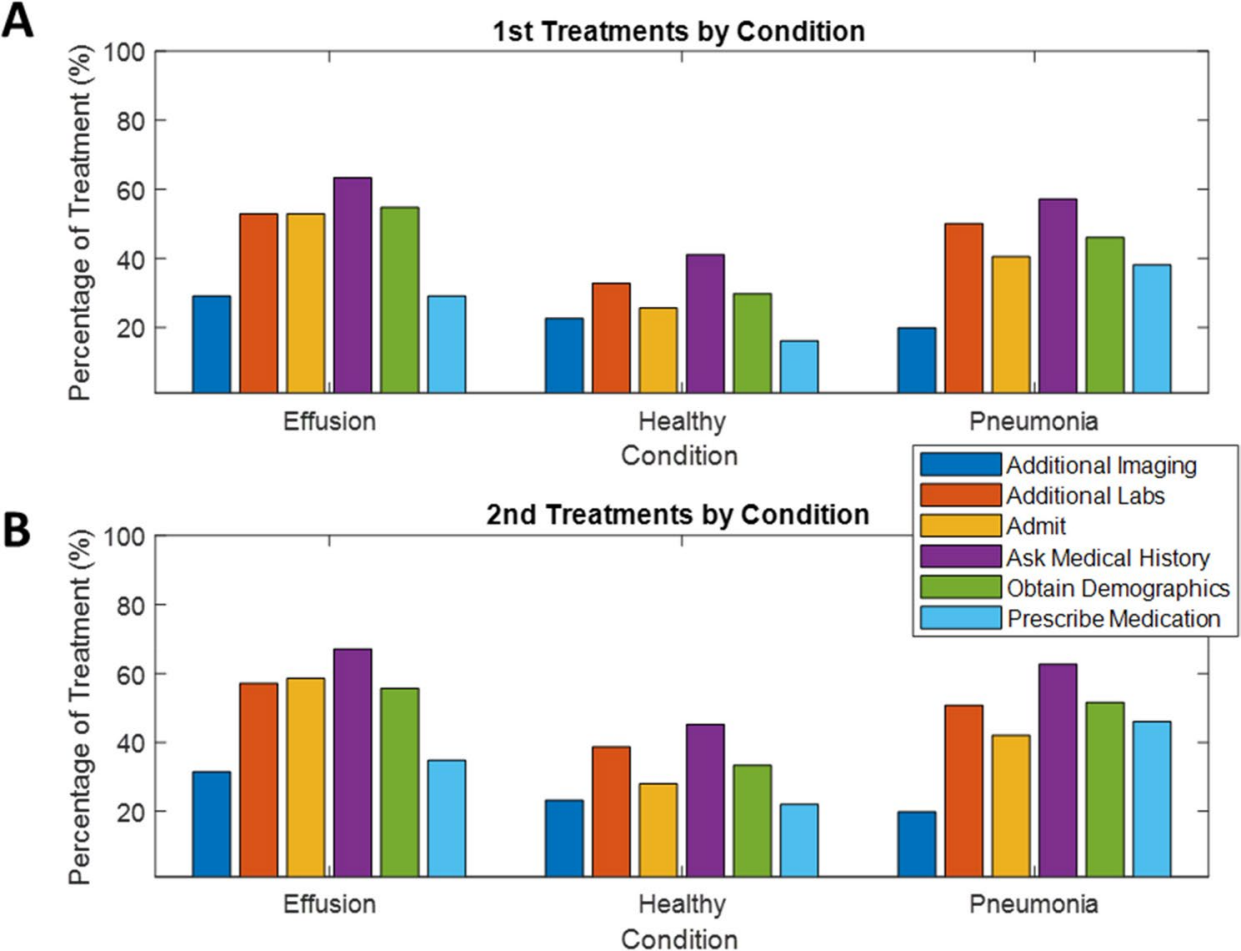
Bar charts depicting the percentage of treatments selected across different conditions. **A** percentage of the trials on which each treatment is selected for each condition before the source (AI-generated or real) is revealed to participants. **B** percentage of the trials on which each treatment is selected for each condition after the source is revealed to participants

For changes in treatment selections (post – pre), the ANOVA showed a significant main effect of Source, (*F*(1, 41) = 13.18, *p* < .001), with more changes following AI-generated images than Real ones. Condition and the interaction were not significant. A separate paired t-test using the binary measure of “any change” confirmed that the likelihood of changing at least one treatment did not differ significantly between Real and AI trials (*t*(41) = 1.10, *p* = .28) (Fig. 8).

Taken together, these findings suggest that participants were better at identifying real images, and felt more confident in their judgements with them, but their treatment plans changed more often when responding to AI-generated images. In addition, increases in confidence were linked to making fewer treatment changes overall, highlighting a disconnect between diagnostic certainty and willingness to revise management decisions.

## Discussion

### Implications

The finding that real images resulted in significantly higher accuracy compared to AI-generated images highlights a key limitation in the current use of AI in radiology education. AI-generated images can sometimes lack the subtle, yet critical, features found in actual patient images, due to imperfect replication of complex nuances of real-world medical images [42, 43]. These discrepancies can lead to clinicians being less confident in diagnoses and may ultimately reduce performance in diagnostic tasks, as we observed here.

However, the introduction of counterfactual images in Block 2 addresses some of these concerns. Since counterfactuals involve manipulating real patient images to reflect different conditions while keeping the underlying anatomical consistency, they provide a more controlled way of simulating various pathological states. While the AI-generated images used in this study are not perfect and may contain subtle inconsistencies, they still offer value in medical education. Despite their imperfections, these images provide a unique opportunity to expose participants to a broader range of scenarios that might be difficult to capture with real patient data alone, thus offering a means to explore rare or complex cases that might not otherwise be available for training [44, 45]. These images simulate realistic diagnostic challenges, encouraging critical thinking and helping to refine the diagnostic skills of clinicians and students [46]. Furthermore, as AI technology advances, the quality of these images is expected to improve, making them an even more powerful tool for training and professional development in the future [47–49].

To optimise the effectiveness of a counterfactual diagnostic training tasks, we suggest incorporating feedback into the existing structure. Previous research has demonstrated that feedback can significantly enhance learning outcomes and diagnostic accuracy by helping students identify conceptual misunderstandings and assess their performance [35, 50]. Previous studies [14, 30, 51] show that repeated exposure to clinical cases improves diagnostic accuracy, supporting the role of accessible feedback in medical education.

One effective method for integrating feedback could involve targeted exercises that highlight distinguishing features of similar conditions. For example, feedback can include actionable insights like: *“Incorrect. For pneumonia*,* look for parenchymal consolidation or increased lung opacity*,* often with patchy or lobar distribution. For pleural effusion*,* focus on the lower lung zones where fluid collects*,* leading to blunting of the costophrenic angle or a homogenous opacity with a meniscus sign.”* Such targeted feedback has been found to significantly improve diagnostic skills and uncertainty when it is timely and relevant [52–55].

Furthermore, in clinical settings, however, providing consistent feedback is often challenging [53, 56, 57]. In contrast, the design of the current tasks allows for easy, consistent feedback delivery, creating a promising opportunity for enhanced learning both medical students and professionals. Moreover, it may be easily delivered online, allowing for the integration of interactive feedback that enhances the learning process [35, 50]. This approach has the potential to become a valuable educational tool, offering a cost-effective and scalable solution that could broaden accessibility for learners without the need for significant resources. By combining evidence-based learning methods with online delivery, it could help radiology education reach a wider audience more efficiently [52, 58] and help reduce unnecessary costs and preventable morbidity [1, 59–61].

Moreover, while our model currently focuses on select condition manipulation, future refinements in image quality could enable more diverse applications, such as extending the included lung-related pathologies and exploring rare or atypical disease presentations by altering demographics like age, gender, or ethnicity. Importantly, X-ray images vary depending on age and gender due to anatomical differences. For instance, older adults often show more subtle or diffuse abnormalities on X-rays because of age-related changes in lung tissue [62–64]. Racial differences also impact the presentation of certain conditions, as some populations are more prone to specific lung diseases, such as tuberculosis in certain ethnic groups [65]. Gender differences, such as lung size and chest wall structures can also influence the appearance of lung pathologies [63, 66]. Thus, there is the potential to ameliorate existing disparities to an extent by creating representative synthetic data that better reflects diverse population [67, 68].

A further limitation concerns the complexity of our study design. By incorporating four distinct but conceptually linked tasks, we aimed to explore different dimensions of learning and decision-making within a single protocol. While this allowed us to capture complementary processes, it also increased participant burden and may have diluted statistical power given the relatively modest sample size (*N* = 42). Future work would benefit from simplifying the design to focus on fewer core tasks with preregistered primary outcomes, while also recruiting larger and more stratified samples to strengthen generalisability. Additional refinements, such as embedding richer clinical context (e.g., demographic details, lab values) and including expert benchmarks, could further enhance ecological validity and trustworthiness of findings.

Evidence suggests that radiologists may hesitate to report rare conditions, even when imaging clearly supports it, due to fear of making errors [19, 69]. As such, we speculate the tool’s potential beyond education, as an interactive resource for practicing clinicians. By enabling doctors to create counterfactuals based on real-time patient data, it could assist in refining diagnoses, exploring differential diagnoses, and providing personalised, context-sensitive learning directly in clinical settings. Additionally, the tool could help overcome diagnostic bias by presenting a variety of counterfactual scenarios that challenge clinicians to consider alternative diagnoses and adjust their decision-making processes, promoting more accurate and objective clinical judgements [67, 68].

To summarise so far, counterfactual chest x-ray images may provide a highly effective resource for radiology education, by providing an interactive, case-based learning experience, which is essential for mastering diagnostic skills in the field [54, 55].

### AI identification

In Block 3, participants were tasked with identifying which of two paired chest X-ray images was real, with one image being altered to show a different condition (e.g., pneumonia, pleural effusion, or healthy). In Block 4, participants were asked to judge whether the image was AI-generated or real, without the benefit of the counterfactual setup. The accuracy scores of 67.4% in Block 3 and 63.7% in Block 4, while moderate, indicate that distinguishing AI-generated images from real ones remains challenging. This is particularly noteworthy given the lack of extensive research on AI identification in medical imaging. Despite the use of counterfactuals in Block 3, the results suggest that even with direct comparisons, accuracy does not reach optimal levels.

The counterfactual images in Block 3 may have provided participants with a more straightforward and potentially less ambiguous task, allowing for clearer distinctions between real and altered images compared to the binary judgement required in Block 4. Thus, having a counterfactual comparison likely made the AI identification task easier by providing a clearer context for participants to judge the differences in conditions.

In this study, participants faced challenges in distinguishing between AI-generated and real images, which is consistent with findings in other fields where high-quality AI images often lead to difficulty in identification [70, 71]. While participants were able to identify AI images on occasion, their ability to do so was not reliable, regardless of whether the measure was based on the slider values or percentage correct. This inconsistency in performance suggests that, despite occasional successes, participants struggled to differentiate AI-generated images from real ones in a dependable manner (see Supplementary Table 2). The difficulty participants experienced in consistently distinguishing between AI-generated and real images suggests that these AI images may be convincing enough for use in educational settings, even if not yet suitable for clinical decision-making. While further validation is needed for clinical applications, the images show sufficient promise to for allowing learners to practice diagnostic skills with diverse cases. While Block 3 performance might suggest limitations in AI image quality, it is important to note that accuracy was only moderate across both real and AI images, indicating that the difficulty may stem more from the task design and lack of clinical context than from image realism alone.

While real images led to higher diagnostic accuracy than AI-generated ones, this discrepancy may reflect limitations in the generative model rather than an inherent shortcoming of counterfactual training. Current AI-generated images can include subtle artefacts that are not present in real images, and the diversity of the real dataset, spanning different patients, imaging systems, and acquisition settings, may also have provided participants with more varied cues. As generative models improve in fidelity and datasets incorporate a wider range of imaging conditions, we expect this performance gap to narrow.

### Treatment management

Participants also selected follow-up actions (such as ordering additional labs, imaging, or prescribing medication) based on their diagnosis. After revealing whether the image was real or AI-generated, participants reassessed their treatment choices and provided updated confidence ratings, allowing for an analysis of how the source of the image impacted their decision-making.

The results further emphasize the importance of building trust in AI-generated images through additional exposure and understanding in medical education [72] summarise various studies evaluating the integration of AI into medical education, shedding light on the current state of AI knowledge, attitudes, and education in the field. These studies, involving medical students, residents, and faculty, highlight a growing interest in AI’s role in healthcare, especially in radiology, though the depth of AI understanding remains limited [73, 74]. A substantial portion of students expressed concerns about AI’s impact on their careers, such as job displacement and decreased interest in specialties like radiology [75, 76]. However, many also acknowledged AI’s potential to improve efficiency, reduce errors, and optimise patient care [77, 78]. As AI continues to transform healthcare, building trust and understanding among medical professionals will be vital. While scepticism exists, the majority of current and future medical professionals recognise AI’s potential to enhance medical practice, emphasising the need for comprehensive training and exposure to AI technologies in medical education [79, 80].

## Limitations and conclusion

A notable limitation of this study is its relatively small sample size, which could influence the generalisability of the findings. Additionally, the sample’s diversity concerning clinical experience and familiarity with AI tools was not extensively detailed, which may restrict the applicability of the findings to broader clinical and educational settings. While participants showed moderate improvements in accuracy and confidence calibration over time, the variability in image quality suggests further refinement of the tool is necessary to maximize its potential.

Furthermore, it is important to note that Block 4 presented imaging in isolation, without access to patient demographics, medical history, or laboratory results. This was an intentional simplification to allow us to isolate the effect of image authenticity (real vs. AI-generated) on confidence and treatment planning. We recognise that this design reduces ecological validity, as clinical decision-making is always informed by wider contextual information. Future work should therefore extend this paradigm to more realistic scenarios that include demographic details, presenting complaints, medical history, and a broader set of treatment options (e.g., discharge with or without antibiotics, pleural aspiration, chest drain insertion, or CT scan) to more closely reflect real-world clinical workflows.

Despite these limitations, the present study highlights the promise of integrating AI-generated images into medical training and practice. The findings demonstrate that participants can achieve moderate diagnostic accuracy and develop better confidence-accuracy alignment with repeated exposure, even when working with mixed real and AI-generated datasets. These results highlight the value of iterative exposure and structured feedback in fostering both diagnostic precision and calibrated confidence.

In conclusion, while the small sample size and controlled conditions temper the generalisability of the results, this study provides foundational evidence supporting the utility of AI-generated images as a training tool. Future research should address these limitations by recruiting larger and more diverse cohorts and exploring more nuanced analyses, such as the effects of varied clinical expertise and experience with AI. With continued refinement of AI tools and expansion of training applications, these technologies hold potential to enhance medical education and clinical decision-making.

## Supplementary Information

Supplementary Material 1.

## Data Availability

Data and materials are available upon reasonable request. Please contact Greta Mohr at [rmjlohr@ucl.ac.uk](mailto: rmjlohr@ucl.ac.uk).

## Abbreviations

AI: Artificial Intelligence
SCM: Structural Causal Model
HAVE: Hierarchical Variational Autoencoder
CXR: Chest X-ray
UCL: University College London
IRB: Institutional Review Board
SD: Standard Deviation
ANOVA: Analysis of Variance
MIMIC-CXR: Medical Information Mart for Intensive Care – Chest X-ray Dataset
MIMIC-IV: Medical Information Mart for Intensive Care – Version IV
EP: Effusion–Pneumonia (trial type)
HE: Healthy–Effusion (trial type)
PH: Pneumonia–Healthy (trial type)
IQR: Interquartile Range
NHS: National Health Service
