# Supplementary material for: Exploring the use of AI-generated counterfactual chest X-rays to enhance diagnostic learning in medical education
Source: BMC Med Educ. 2026 Jun 12;26:1331. doi: 10.1186/s12909-026-09597-7 (PMC13483552; doi:10.1186/s12909-026-09597-7)
Supplement: Supplementary file 1 [file 12909_2026_9597_MOESM1_ESM.docx]

# Supplementary Material

## Example Stimuli

Figure S1. Example stimuli showing the original x-ray images and the subsequent counterfactual images generated to show alternative conditions.

## Decision Strength

We compared the decision strength and confidence between correct (>55) and incorrect (<45) trials. Decision strength was calculated by measuring the distance between the participant's slider response and the maximum value of the slider (100). For correct trials, this distance represented how close the participant's response was to the correct answer. For incorrect trials, the score was reversed, meaning that the distance reflected how far the response was from the perceived correct answer.

| Block 1 | | | | |
| --- | --- | --- | --- | --- |
|  | First Half (pre) | | Second Half (post) | |
|  | Strength | Confidence | Strength | Confidence |
| Correct | 91.0 | 4.8 | 9.7 | 4.6 |
| Incorrect | 89.7 | 4.6 | 88.0 | 4.2 |
| Uncertain | 50.2 | 2.8 | 48.6 | 3.2 |
| Block 2 | | | | |
|  | First Half | | Second Half | |
|  | Strength | Confidence | Strength | Confidence |
| Correct | 91.7 | 4.8 | 90.9 | 4.8 |
| Incorrect | 90.5 | 4.3 | 90.6 | 4.4 |
| Uncertain | 50.2 | 4.5 | 51.0 | 3.1 |
| Block 3 | | | | |
|  | First Half | | Second Half | |
|  | Strength | Confidence | Strength | Confidence |
| Correct | 91.4 | 4.6 | 89.9 | 4.6 |
| Incorrect | 88.7 | 4.1 | 88.5 | 3.9 |
| Uncertain | 48.8 | 2.0 | 50.5 | 2.9 |
| Block 4 | | | | |
|  | First Half | | Second Half | |
|  | Strength | Confidence | Strength | Confidence |
| Correct | 88.8 | 4.2 | 88.2 | 4.4 |
| Incorrect | 88.3 | 4.2 | 86.6 | 4.1 |
| Uncertain | 49.3 | 2.8 | 49.6 | 3.3 |

Table S1.Comparison of decision strength and confidence for different conditions (Correct, Incorrect, and Uncertain) across the first and second halves of four blocks. The table shows the strength of decisions (distance from the correct answer) and the reported confidence for each trial type (Correct, Incorrect, and Uncertain).

The data across all four blocks shows consistent trends in decision strength and confidence levels across trial types (correct, incorrect, and uncertain) for both halves of the task. For decision strength, correct trials consistently exhibit the highest values compared to the incorrect and uncertain trials. In each block, the decision strength for correct trials tends to decrease slightly from the first half (pre) to the second half (post), although the change is relatively modest. This trend suggests that participants may become slightly less confident or decisive after repeated exposure to the task, even when they are making correct judgments.

Incorrect trials show a similar pattern, with decision strength slightly decreasing in the second half of each block. The confidence levels for incorrect trials also follow this trend, with a noticeable decline in the second half. This suggests that participants' confidence in their incorrect decisions diminishes as they progress through the task, possibly due to increased recognition of errors or difficulties in making correct judgments. Uncertain trials, defined as those with decision strengths between 45 and 55, naturally exhibit medium decision strength so is unsurprising as these trials represent situations where participants are uncertain about the correct answer. Interestingly, the confidence levels for uncertain trials are lower than those for correct and incorrect trials, with minimal variation between the first and second halves of the block. This suggests that participants had reduced confidence in their judgments during uncertain trials, aligning with the difficulty these trials present. Overall, the trends across all four blocks suggest that decision strength and confidence are highest for correct trials, slightly decreasing as participants encounter more challenging or incorrect trials. The results highlight how decision-making and confidence evolve as participants engage with more difficult or ambiguous tasks, with notable declines in confidence for incorrect and uncertain trials, especially in the latter half of each block.

## Comparing Accuracy Measures

In this study, the Slider measure was used in the main analysis for richer data, offering a more nuanced understanding of participants’ precision. By comparing these two methods, we can gauge how well the Slider method captures more subtle variations in diagnostic performance, as compared to the more binary Percentage Correct measure, which simply quantifies accuracy. Both measures displayed similar trends across the blocks, but this comparison highlights the strengths of using the Slider for more detailed insights while also validating the simpler Percentage Correct measure as a reliable, though less granular, metric.

|  | Slider | | Percentage | |
| --- | --- | --- | --- | --- |
|  | First Half | Second Half | First Half | Second Half |
| Block 1 | 61.9 | 63.3 | 64.1 | 64.6 |
|  |  |  |  |  |
|  |  |  |  |  |
| Block 2 | 63.6 | 70.4 | 65.5 | 74.1 |
|  |  |  |  |  |
|  |  |  |  |  |
| Block 3 | 69.5 | 65.4 | 72.0 | 67.7 |
|  |  |  |  |  |
|  |  |  |  |  |
| Block 4 | 69.1 | 58.3 | 72.2 | 59.1 |
|  |  |  |  |  |
|  |  |  |  |  |

Table S2. Comparison of task performance measured by two methods: Slider Accuracy (based on the distance from the correct answer on a scale with negative marking) and Percentage Correct (based on the number of trials answered correctly).

Across the four blocks, both the Slider accuracy and Percentage Correct showed similar trends, with improvements in Block 1 and Block 2, and declines in Block 3 and Block 4. However, the Percentage Correct measure generally yielded higher values compared to the Slider accuracy. Despite this, both measures followed the same pattern of increase in Block 1 and Block 2, and decrease in Block 3 and Block 4.

## Confidence and Accuracy Across Experience Levels

Next, we looked at the comparison of confidence and accuracy between medical students and doctors across four distinct blocks of a medical imaging task. This analysis aimed to identify differences in performance between the two groups, as well as explore the potential impact of years of experience on both confidence and diagnostic accuracy. T-tests were conducted to compare confidence and accuracy between medical students and doctors, while Pearson correlations were calculated to assess the relationship between years of experience and these performance metrics. The results provide insights into how experience may influence diagnostic confidence and accuracy in medical imaging tasks.

| Means | | | | |
| --- | --- | --- | --- | --- |
|  | Medical Student | | Doctor | |
| Block | Confidence | Accuracy | Confidence | Accuracy |
| One Pre | 4.6 | 62.5 | 4.9 | 60.7 |
| OnePost | 4.4 | 62.4 | 4.7 | 64.7 |
| Two | 4.6 | 67.2 | 4.8 | 66.7 |
| Three | 4.3 | 66.8 | 4.5 | 68.5 |
| Four | 4.1 | 63.4 | 4.4 | 64.1 |
|  |  |  |  |  |
| Years Experience - Pearson Correlation | | | | |
|  | Confidence | | Accuracy | |
| Block | Correlation | P-Value | Correlation | P-Value |
| One Pre | 0.02 | 0.56 | -0.05 | 0.21 |
| OnePost | 0.01 | 0.69 | 0.00 | 0.90 |
| Two | -0.04 | 0.34 | 0.03 | 0.43 |
| Three | -0.11 | 0.00 | 0.02 | 0.49 |
| Four | -0.17 | 0.00 | -0.07 | 0.11 |
|  |  |  |  |  |
| T-Tests | | | | |
|  | Confidence | | Accuracy | |
| Block | T Stat | P-Value | T Stat | P-Value |
| One Pre | -3.07 | 0.00 | 0.57 | 0.57 |
| OnePost | -2.94 | 0.00 | -0.79 | 0.43 |
| Two | -2.45 | 0.01 | 0.15 | 0.88 |
| Three | -1.94 | 0.05 | -0.68 | 0.49 |
| Four | -2.03 | 0.04 | -0.21 | 0.83 |

Table S3. Table summarizing the comparison between medical students and doctors across four blocks, showing their confidence and accuracy scores along with correlations to years of experience.

To summarise, these results suggest that while medical students and doctors differed slightly in confidence, their accuracy remained relatively comparable across blocks, with medical students showing some improvement in accuracy over time. Experience, as indicated by years of training, had little to no impact on accuracy, but a minor negative correlation with confidence, especially in later blocks.
